# Effect of a multistrain probiotic (Lactoflorene^®^ Plus) on inflammatory parameters and microbiota composition in subjects with stress-related symptoms

**DOI:** 10.1016/j.ynstr.2018.11.001

**Published:** 2018-11-07

**Authors:** Sara Soldi, Sara Carlotta Tagliacarne, Chiara Valsecchi, Simone Perna, Mariangela Rondanelli, Luigi Ziviani, Stefano Milleri, Ariella Annoni, Annamaria Castellazzi

**Affiliations:** a AAT – Advanced Analytical Technologies Srl, via P. Majavacca 12, 29017, Fiorenzuola d’Arda, Pc, Italy; b Department of Clinical Surgical Diagnostic and Pediatric Sciences, University of Pavia, P.le Golgi 2, 27100, Pavia, Italy; c Department of Pediatrics, Fondazione IRCCS Policlinico San Matteo, 27100, Pavia, Italy; d Department of Biology, College of Science, University of Bahrain, Sakhir Campus, P. O. Box 32038, Bahrain; e Department of Public Health, Experimental and Forensic Medicine, School of Medicine, Endocrinology and Nutrition Unit, University of Pavia, Pavia, Italy; f Centro Ricerche Cliniche di Verona Srl, P.le L.A. Scuro 10, 37134, Verona, Vr, Italy; g Montefarmaco OTC, via IV Novembre 92, 20021, Bollate, Mi, Italy; h IRCCS Mondino Foundation, Pavia, Department of Public Health, Experimental and Forensic Medicine, Unit of Human and Clinical Nutrition, University of Pavia, Italy

**Keywords:** BB-12^®^, LA-5^®^, Immune response, Stress, Abdominal pain, IgA

## Abstract

Stress affects the immune system and intestinal microbiota composition and can lead to imbalance between pro- and anti-inflammatory cytokines or to uncontrolled production of cytokines. The effect of emotional stress on secretory IgA levels also indicates that stress decreases mucosal integrity. Our aim was to evaluate whether a probiotic product (Lactoflorene^®^ Plus) can prevent alterations in the immune response associated with self-reported stress and microbiota composition. Healthy adult volunteers who self-reported psychological stress were enrolled and randomised into a placebo and a probiotic group. Salivary stress markers (α-amylase, cortisol, chromogranin A) and immunological parameters (sIgA, NK cell activity, IL-8, IL-10, TNF-α) in feces and the composition of intestinal microbiota were evaluated. Administration of the product did not exert a direct effect on the salivary stress markers or NK cell activity but did reduce abdominal pain and increase faecal IgA and IL-10 levels. The probiotic product induced a moderate increase in *Bifidobacterium* and *Lactobacillus* spp., as expected, and in *Faecalibacterium* spp., and decreased the size of the *Dialister* spp. and *Escherichia* and *Shigella* populations. Administration of the product helped protect the mucosal barrier by supporting the number of short-chain fatty acid producers and decreasing the load of potentially harmful bacteria, thus reducing intestinal inflammation and abdominal discomfort.

**ClinicalTrials.gov:**

NCT03234452.

## 1 Introduction

A significant increase in emotional health issues, such as depression, stress and anxiety, has been recently reported (Bressert, 2006; Kessler and Greenberg, 2009). Stress and anxiety are common feelings, with stress being a response to a situational threat while anxiety is a reaction to that stress.

It is difficult to estimate the economic burden of anxiety disorders, but they were calculated to cost the US economy $47 billion in 2000 (Kessler and Greenberg, 2009). Consequently, stress and related conditions are the focus of increased interest and are the subject of new therapies based on traditional and alternative medicines (Asher et al., 2017).

There is evidence that stress induces gut microbial dysbiosis and resultant bowel dysmotility, inflammation and increased permeability, including a substantial decrease in beneficial bacteria such as *Lactobacilli* and *Bifidobacteria* and an increase in potentially pathogenic microorganisms such as *Escherichia coli* (Hawrelak and Myers, 2004).

Intestinal bacteria contribute to host metabolism, for example by producing metabolites such as short-chain fatty acids (SCFAs) which have neuroactive properties (Dinan et al., 2015; Russell et al., 2013). Some species of *Lactobacillus, Bifidobacterium*, *Escherichia* and *Bacillus* can also produce neurotransmitters and neuromodulators such as γ-aminobutyric acid, norepinephrine and dopamine as reported by Lyte (2011, 2013) and Wikoff et al. (2009). Moreover, some probiotics can also modulate opioid and cannabinoid receptors in gut epithelium (Rousseaux et al., 2007). Emerging evidence suggests that the gut microbiome can influence the core symptoms of neuropsychiatric disorders, such as stress, depression and anxiety, giving rise to the concept of ‘psychobiotics’, which are defined as probiotics that, when ingested in appropriate quantities, confer mental health benefits (Dinan et al., 2013).

Studies in Sprague–Dawley rats have demonstrated a link between the gut microbiota and depressive-like behaviour (Abildgaard et al., 2017). Similarly, Neufeld McVey et al. (2017) demonstrated that a diet containing a mixture of prebiotic molecules and probiotic bacteria administered to rats was able to reduce the anxiety-like behaviour caused by early-life separation. Another intriguing finding was reported by Vanhaecke et al. (2017) in a study on new-born rats with verified intestinal barrier integrity and permeability who were receiving *Lactobacillus fermentum* CECT 5716. Positive results were also documented by Cowan et al. (2016) and Takada et al. (2016), confirming that administration of *Lactobacillus casei* Shirota fermented milk or of a mixture of *Lactobacillus rhamnosus* and *L. fermentum* could relieve long-term psychological and physiological stress-associated symptoms. However, despite the positive results found in animal models, clinical translation to humans is still in uncertain.

Kelly et al. (2017) recently conducted a study to confirm in human volunteers the promising results of preclinical tests conducted in an anxious mouse model. On the other hand, Allen et al. (2016) tested a potential psychobiotic, *Bifidobacterium longum* 1714, for its impact on stress-related behaviours, physiology and cognitive performance with very encouraging results.

In line with the increased attention given to stress and to the treatment of mental disorders with non-pharmacological approaches, other authors have recently studied psychobiotic administration with varied results (Akkasheh et al., 2016; Bambling et al., 2017; Cepeda et al., 2017; Colica et al., 2017; Steenbergen et al., 2015). A frequent and interesting finding was that probiotic strains showed an anti-inflammatory effect due to increased production of anti-inflammatory cytokines or a reduction in gut barrier dysfunction (Bambling et al., 2017; Nishida et al., 2017; Vanhaecke et al., 2017).

Natural killer (NK) cell activity is affected by stress, with its alteration linked to both environmental signals and mental health. Subjects who have experienced acute emotional stress show a marked reduction in NK cell activity and *in vitro* response to antigens (Boscolo et al., 2009, 2012; Duggal et al., 2015; Geiger and Sun, 2016; Kim et al., 2017; Morikawa et al., 2005).

Many parameters may be affected by stress and related symptoms, although the mechanisms are not yet fully understood. Evaluable hormonal markers of stress in the saliva include cortisol (He et al., 2010; Thatcher et al., 2004), α-amylase (SAA) and chromogranin A, also present in other biological fluids such as plasma and serum (Filaire et al., 2009).

Cytokines, such as tumour necrosis factor-α, IL-8 and IL-10, play a central role in modulation of the intestinal immune system and probiotic microorganisms could have an influence on their activation. (Dinan et al., 2013).

IgA as well plays a fundamental role in the defence against pathogenic organisms by inhibiting bacterial adherence and promoting their elimination from the gastrointestinal tract (Bunker et al., 2017; Campos-Rodríguez et al., 2013).

Human studies have shown that acute stress may increase intestinal permeability, which is related to a number of diseases including gastrointestinal disorders such as inflammatory bowel disease (Crohn's disease and ulcerative colitis), coeliac disease and irritable bowel syndrome (Dunlop et al., 2006; Gecse et al., 2012).

The aim of the present study was to explore the ability of a multistrain probiotic mixture to modulate inflammatory markers, intestinal barrier function and gastrointestinal symptoms in healthy volunteers with self-reported anxiety as an indirect indication of perceived stress. The tested bacterial mixture contained in the product (*Lactobacillus acidophilus* LA-5^®^, *Bifidobacterium animalis* subsp. *lactis*, BB-12^®^, *Lactobacillus paracasei* subsp. *paracasei*, L. CASEI 431^®^, *Bacillus coagulans* BC513) was selected based on their characteristics *in vitro* with respect to acid- and bile tolerance, adhesion and the inflammatory response.

Strains were also selected on the basis of trans-epithelial resistance (TER), which is an indicator of the ability of the probiotic strains to increase the tight junction barrier between cells in a model of the gastrointestinal barrier. The inflammatory response was assessed as the IL-10/IL-12 ratio induced in human, isolated white blood cells (unpublished data, Chr. Hansen).

## 2 Methods

This study was a randomised, double-blind, placebo-controlled, cross-over trial performed at a single centre (Centro Ricerche Cliniche di Verona, CRC) in Italy between 23 March 2016 and 21 July 2016.

The study was performed in accordance with the principles of the Declaration of Helsinki, good clinical practice and applicable national regulatory requirements. All procedures involving human subjects were approved by the Ethical Committee for Clinical Trials of the Provinces of Verona and Rovigo. Written informed consent was obtained from all subjects before enrolment. The study was registered at ClinicalTrials.gov under registration number NCT03234452.

### 2.1 Study participants

The recruiting strategy involved searching the already populated CRC healthy volunteer database and increasing public visibility by announcing the study on the online CRC site and by posting advertisements and flyers. Eligible subjects were healthy men and women, 20–35 years old, with a State-Trait Anxiety Inventory (STAI) scale, form Y, module 1 (state anxiety) score of ≥35 for men and ≥40 for women. The most important exclusion criteria were a history or diagnosis of gastrointestinal disease, oral antibiotics within 30 days before the screening visit, and use of drugs, food or herbal supplements for digestive symptoms. A complete list of the inclusion and exclusion criteria is provided in the supplementary file.

### 2.2 Study design

The study was conducted with a cross over design with the primary aim to reduce the influence of confounding covariates because patients can serve as their own control and, additionally, because of the efficiency linked to this kind of designs, requiring fewer subjects than do non-crossover studies (Jones and Kenward, 2003).

At the screening visit, eligible subjects were enrolled in a 2-week run-in period to wash out potential pre-study probiotics. The run-in period was followed by two treatment periods (probiotic and placebo) of 45 days each separated by a wash-out period of 25 days. The total length of the study was 129 days. Subjects with state anxiety as determined by scores on the State-Trait Anxiety Inventory (STAI) form Y, module 1 were included. Efficacy was evaluated at each of four visits: visit 2 (start of first intervention, day 0), visit 3 (end of first intervention, day 45), visit 4 (start of second intervention, day 70) and visit 5 (end of second intervention, day 115). Saliva, blood and faecal samples were collected at visits 2, 3, 4 and 5.

Information on gastrointestinal symptoms (abdominal pain, aerophagia, diarrhea, constipation and alternating diarrhea/constipation) was collected at visits 4 and 5 using a modified version of the Subjective Health Complaints Inventory.

### 2.3 Randomisation and treatment

A randomisation list was generated using the web program List Randomizer from https://www.random.org. Randomisation to the two groups was performed in a 1:1 ratio and study products were only identified by their randomisation number. The investigator allocated subject consecutively by assigning the first available randomisation number to eligible subjects. All subjects, investigators, the contract research organisation (CRO) and sponsor staff involved in the study were blinded until the final database was locked. Only staff at the CRO and the sponsor, who were not otherwise involved in the study, had access to the randomisation list so they could label the study products.

Subjects were randomly assigned to receive 10 ml of a liquid mixture of either *Lactobacillus acidophilus* LA-5^®^, *Bifidobacterium animalis* subsp. *lactis*, BB-12^®^, *Lactobacillus paracasei* subsp. *paracasei*, L. CASEI 431^®^, *Bacillus coagulans* BC513, zinc and B vitamins (niacin, B1, B2, B5, B6, B12 and folic acid) with 2 billion CFU/10 ml (Lactoflorene^®^ Plus), or an identical placebo liquid mixture containing no probiotics. The active and placebo products were produced by Biofarma (Italy), had a similar appearance, taste and smell and were provided in identical 10 ml bottles with identical labelling. Subjects consumed two 10 ml bottles per day. Compliance was evaluated at the end of each treatment period, when the subjects were asked to return all treatment bottles and the numbers of used and unused bottles were counted.

### 2.4 Outcome measures

The primary endpoint was NK cell activity, which was measured in a sample of approximately 5 ml of whole blood taken at set times from all participants, using heparinised tubes.

Cortisol, α-amylase and chromogranin A were quantified in saliva collected from each participant in the morning in 15 ml tubes: samples were taken at least 6 h after eating, before smoking, coffee consumption or brushing the teeth.

Faecal IgA, IL-8, TNF-α and IL-10 were measured in faecal water extracted from frozen stool samples collected at scheduled times in disposable containers. The microbiota composition of the same samples was also investigated after total bacterial DNA extraction.

### 2.5 Primary endpoint

The primary endpoint was NK cell activity defined as an effector:target ratio of 100:1. Significantly higher concentrations of NK cell activity were expected from the group taking the probiotic compared with the placebo group.

### 2.6 Secondary endpoints

Secondary endpoints included immunological markers quantified in saliva, stool and blood: in the probiotic group, cortisol, α-amylase and chromogranin A levels in saliva were expected to decrease, while IgA levels in stool samples were expected to increase, as was IL-10. On the other hand, a reduction in IL-8 and TNF-α was expected in the probiotic group compared with placebo. Changes in microbiota composition were also investigated using NGS technology: the probiotic product was expected to have an effect due to an increase in the *Lactobacilli* and *Bifidobacteria* administered with the product, thus improving the protective function of the gut mucosa, which is frequently impaired by stress conditions.

Gastrointestinal symptoms were assessed in both groups using a questionnaire at the beginning and end of the second treatment period. Symptoms were expected to improve in the probiotic group, with a decrease in constipation, diarrhea, abdominal pain and aerophagia after supplementation.

Data on adverse events (AE) were gathered from the beginning of treatment to the end of the study. AEs were collected at each study visit by asking the subjects the non-leading question ‘Have you had any health problems since the previous visit/you were last asked?’. Subjects were also free to report AEs at any time during the study. An AE was defined as any untoward medical occurrence in a subject administered the study product, whether or not considered related to the study treatment. The study investigator rated the relatedness of any AE to the study product.

### 2.7 Statistical analysis

#### 2.7.1 Sample size

The sample size calculation was based on a two-sided α level of 0.05, SD of 0.6 and 80% power to detect a potential effect of the product on NK cell activity. To allow for a potential 10% drop-out rate, 25 subjects in each group were needed, giving a total of 50 subjects (Reale et al., 2012).

#### 2.7.2 Statistical analysis of questionnaires and immunological output

A two-sided p value of 0.05 was considered to be significant. Continuous variables were summarised, by treatment group, into their means and SD.

For each outcome, paired t-tests were used to evaluate variations within treatment from baseline (t_0_) to the end of follow-up (t_1_) in both the placebo and the intervention group.

Linear regression models, adjusted for age, sex and STAI score at baseline, were used to obtain β (Δ-change differences in treatment versus placebo) over time (i.e., mean differences between treatments over time).

All p values were two-tailed and considered to be statistically significant if p < 0.05. Statistical analyses were performed using IBM^®^ SPSS^®^ statistics for Windows 21.0.0.0 (IBM Corp.).

### 2.8 Sample collection and analysis

#### 2.8.1 Saliva samples

Saliva samples were centrifuged at 13000 rpm for 1 min and supernatants were collected and stored at −20 °C until use. Cortisol, α-amylase and chromogranin A levels were evaluated using ELISA kits. Salivary IgA was also detected with an ELISA test as previously described (Avanzini et al., 1992).

#### 2.8.2 Faecal samples

Faecal supernatants for cytokine evaluation were extracted using a procedure modified from Nielsen and Jahn (1999)*.* Faecal supernatants were obtained after resuspension of a small amount of faecal sample in PBS buffer (1:2 wt:volume ratio, g/ml) and centrifugation at 13000 rpm for 1 min. The levels of IL-8, IL-10, TNF-α and IgA were measured using ELISA tests (Avanzini et al., 1992).

#### 2.8.3 Isolation of peripheral blood mononuclear cells from whole blood

For the isolation of peripheral blood mononuclear cells (PBMC), blood samples were centrifuged on Ficoll-Hypaque density gradient medium and resuspended in complete RPMI 1640 medium (Euroclone, Italy), supplemented with heat-inactivated 10% FCS (Euroclone), 2.5 mmol/l l-glutamine (Gibco) and 50 μg/ml gentamycin (Gibco).

Cells were then resuspended in FCS+10% DMSO (Edwards Lifesciences) and cryopreserved in a programmable controlled-rate cell freezer.

#### 2.8.4 Quantification of sIgA levels in saliva samples and IgA in faecal supernatants

96-well microtiter plates (Greiner) were coated with polyclonal rabbit anti-human IgA (Dako) and incubated for 3 h at 37 °C and then overnight at 4 °C. On the second day, saliva samples or faecal supernatants were incubated for 2 h at 37 °C and then secondary rabbit anti-human IgA antibody conjugated to horse-radish peroxidase (HRP) (Dako) was added. The plate was read at 492 nm in a Sunrise microplate reader (Tecan) and the concentration of sIgA was extrapolated from a standard curve included in each plate and expressed in ng/ml.

#### 2.8.5 Quantification of salivary stress markers

Cortisol, α-amylase and chromogranin A in saliva samples were evaluated using ELISA kits.

#### 2.8.6 Quantification of cytokine levels in faecal supernatants

Microtiter plates were briefly coated with purified monoclonal antibody anti-human IL-10, IL-8 and TNF-α (Endogen-Tema, USA). After stabilisation with 2% BSA (Sigma) in PBS (Euroclone) for 1 h, the samples were added. Monoclonal anti-human biotinylated antibody IL-10, IL-8 and TNF-α were used. The reproducibility and specificity of the assay had been verified previously. The plate was read at 450 nm in a Sunrise microplate reader (Tecan) and the concentration of cytokines was extrapolated from a standard curve included in each plate and expressed in ng/ml.

#### 2.8.7 NK cell activity

The PBMC of the probiotic and placebo groups were isolated from whole blood after centrifugation on a Ficoll-Hypaque gradient. NK cytotoxic activity was evaluated after incubation of PBMC with K562 cells (human chronic myelogenous leukaemia cells) previously labelled with ^51^Cr (Perkin Elmer, USA). The PBMC and K562 cells were incubated in RPMI 1640 + 10% FCS for 4 h. NK cell activity was determined as the percentage of K562 lysed by NK cells, and was evaluated with a TOPCount counter (Packard).

#### 2.8.8 Microbiota identification by 16S rRNA gene amplification, sequencing and data analysis

A stool sample consisting of 6–10 g of fresh faecal material was obtained from each subject and cooled to 4 °C immediately after collection. All samples were then transferred to GenProbio Srl (Probiogenomics Laboratory, University of Parma) and maintained at −80 °C until processing. DNA was extracted from each stool sample using the QIAamp DNA Stool Mini kit following the manufacturer's instructions (Qiagen).

Partial 16S rRNA gene sequences were amplified from extracted DNA using primer pair Probio_Uni/Probio_Rev, which targets the V3 region of the 16S rRNA gene sequences (Milani et al., 2013). 16S rRNA gene amplification and amplicon checks were then carried out as previously described (Milani et al., 2013). 16S rRNA gene sequencing was performed using a MiSeq sequencer (Illumina) at the DNA sequencing facility of GenProbio Srl according to the protocol previously reported (Milani et al., 2013). After sequencing, the individual sequence reads obtained were filtered using Illumina software to remove low-quality and polyclonal sequences. All Illumina quality-approved, trimmed and filtered data were exported as.fastq files which were processed using a custom script based on the QIIME software suite (Caporaso et al., 2010). The sequence data have been submitted to the GenBank databases under accession number SRP126232.

## 3 Results

**Fig. 1:**
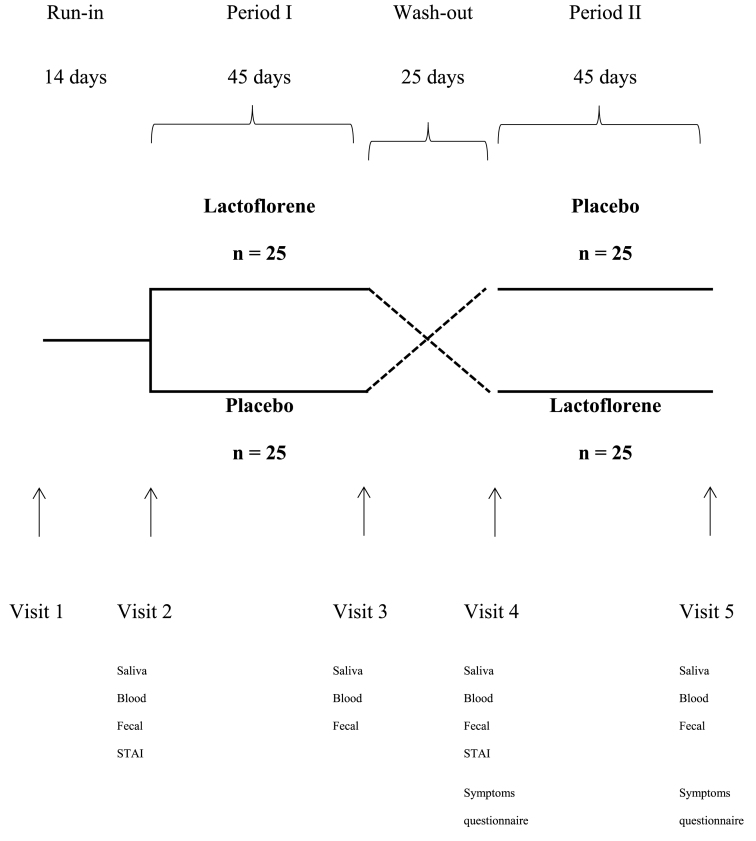
Study design scheme with all the samples provided by subjects for each timepoint.

Fifty subjects were eligible to participate in the study and were randomised into a probiotic and placebo group. Three subjects discontinued the study: two withdrew because of inability to collect the last sample of the second treatment period and one because of a need to take antibiotics during the wash-out period. A flowchart of participant involvement is shown in Fig. 1. The characteristics of the two study groups were similar at baseline (Table 1). Regarding the carry-over effect, although the wash-out period lasted 25 days, we found significant differences in NK cells (p = 0.014) and in cortisol (p = 0.025) between the two sets of baseline data.

**Table 1:** Baseline characteristics of the study population.

Characteristics	Intervention (n̂25)	Placebo (n̂24)	p-value
Aerophagia (SCORE)	0.04 (0.95)	0,04 (0,47)	0.99
Diarrhea (SCORE)	0.08 (0.41)	−0.04 (0.71)	0.45
Costipation (SCORE)	0.00 (0.83)	−0.30 (0.88)	0.29
Abdominal pain (SCORE)	0.00 (0.66)	0.09 (0.29)	0.56
Diarrhea-Costipation ((SCORE)	- 0.08 (0.28)	0.09 (0.52)	0.16
sIgA (μg/ml)	137.52 (70.87)	149.64 (109.46)	0.47
sAA (ng/gr of feces)	59.1 (30.19)	84.18 (25.41)	**p**<**0,05**
CgA (pg/gr of feces)	1848.19 (1027.56)	1366.36 (786.22)	0.31
Cortisol (ng/gr of feces)	5.72 (2.39)	5.87 (2.61)	0.77
IgA (μg/gr of feces)	30.29 (35.64)	50.91 (142.34)	0.35
IL8 (pg/gr of feces)	0.29 (1.02)	0.24 (1.05)	0.81
IL10 (pg/gr of feces)	12.57 (16.20)	40.90 (80.42)	**p**<**0,05**
TNFa (pg/gr of feces)	0.96 (2.43)	1.79 (3.49)	0.21
NK (%lysis)	7.08 (8.77)	4.90 (6.21)	0.67

Bold characters highlight the statistically different values obtained from parameters investigation.

### 3.1 Primary efficacy analysis

NK cell activity did not show any significant variation in the intervention group, as shown in Table 2. Three different target: effector ratios were used and the same behaviour was observed in all three cases. NK cell activity was slightly lower in the probiotic group than in the placebo group: NK 1:10, Δ = −1.25; p = 0.21; NK 1:30, Δ = −0.86; p = 0.51; and NK 1:100, Δ = −1.70; p = 0.34.

**Table 2:** Effect of treatment versus placebo on variables.

Variable (unit)	Intervention				Placebo			
	Baseline	After 45 days	Δ change (pre-post)	p-value	Baseline	After 45 days	Δ change (pre-post)	p-value
Aerophagia (SCORE)	0.04 (0.95)	0.08 (0.58)	0.04 (−0.53; 0.45)	0.86	0,04 (0,47)	0.00 (0.30)	−0,04 (−0.2; 0.3)	0.71
Diarrhea ((SCORE)	0.08 (0.41)	0.17 (0.38)	0.08 (−0.33; 0.16)	0.49	−0.04 (0.71)	0.09 (0.60)	0,13 (−0.5; 0.22)	0.45
Constipation ((SCORE)	0.00 (0.83)	0.00 (0.66)	0.00 (−0.47; 0.47)	1	−0.30 (0.88)	0.04 (0.64)	0,35 (−0.9; 0.16)	0.18
Abdominal pain ((SCORE)	0.00 (0.66)	−0.17 (0.76)	−0.17 (0.28; 0.61)	0.45	0.09 (0.29)	0.09 (0.29)	0 (−0.13; 0.13)	1
Diarrhea-Costipation (SCORE)	- 0.08 (0.28)	−0.08 (0.50)	0.00 (−0.25; 0.25)	1	0.09 (0.52)	−0.04 (0.48)	−0.13 (−0.17; 0.43)	0.38
sIgA (μg/ml)	137.52 (70.87)	149.43 (120.70)	11.91 (−41.17; 17.35)	0.42	149.64 (109.46)	162.06 (146.73)	12,41 (−38.9; 16.07)	0.35
sAA (ng/gr of feces)	59.1 (30.19)	75.31 (42.89)	16.21 (−52.87; 20.45)	0.32	84.18 (25.41)	88.42 (29.46)	4.24 (−17.27; 8.80)	0,50
CgA (pg/gr of feces)	1848.19 (1027.56)	2138.72 (1148.22)	290.52 (−806.74; 225,69)	0.26	1366.36 (786.22)	1702.18 (1083.46)	335,82 (−765.27; 93.62)	0.12
Cortisol (ng/gr of feces)	5.72 (2.39)	5.59 (3.12)	−0.13 (−24.50; 6.88)	0.76	5.87 (2.61)	5.42 (2.47)	−0.45 (−22.14; 64.8)	0.13
IgA (μg/gr of feces)	30.29 (35.64)	39.10 (40.26)	8.81 (−0.7; 0.25)	0.27	50.91 (142.34)	29.58 (33.42)	−21.33 (−0.2; 0.15)	0.33
IL8 (pg/gr of feces)	0.29 (1.02)	0.51 (1.28)	0.22 (−21.47; 2.57)	0.35	0.24 (1.05)	0.26 (1.13)	0.02 (−33; 31.77)	0.79
IL10 (pg/gr of feces)	12.57 (16.20)	22.02 (35.58)	9.45 (−1.10; 0.46)	0.12	40.90 (80.42)	41.75 (111.60)	0.85 (−0.94; 1.55)	0.96
TNFa (pg/gr of feces)	0.96 (2.43)	1.72 (3.10)	0.77 (−0.68; 2.06)	0.22	1.79 (3.49)	1.49 (2.77)	−0.30 (−1.77; 0.75)	0.63
NK 1:10 (%lysis)	4.08 (4.84)	3.39 (6.10)	−0.69 (−0.66; 2.67)	0.32	2.43 (3.04)	2.95 (3.71)	0.51 (−2.47; 1.69)	0.42
NK 1:30 (%lysis)	7.08 (8.77)	6.08 (8.09)	−1 (−1.07; 3.10)	0.23	4.90 (6.21)	5.28 (5.67)	0.39 (−2.53; 3.8)	0.71
NK 1:100 (%lysis)	11.12 (12.48)	9.67 (9.70)	−1.45 (−4.42; 3,32)	0.25	9.94 (10.20)	9.31 (8.95)	−0.63 (−9.23; 8.34)	0.69

### 3.2 Secondary efficacy analysis

When differences between the data before and after the intervention were examined, the salivary stress markers did not display any significant variations between the probiotic and placebo groups: sIgA +11.91 (−41.17, 17.35; p = 0.42) for intervention subjects versus +12.41 (−38.9, 16.07; p = 0.35) for placebo subjects; α-amylase +16.21 (−52.87, 20.45; p = 0.32) versus 4.24 (−17.27, 16.07; p = 0.35); chromogranin A 290.52 (−806.74, 225.69; p = 0.26) versus 335.82 (−765.27, 93.62; p = 0.12); and cortisol −0.13 (−24.50; 6.88; p = 0.76) versus −0.45 (−22.14, 64.8; p = 0.13). The results showed significant individual variability.

As reported in Table 2, faecal IgA showed an increase in the intervention group compared with placebo: IgA +8.81 (−0.7, 0.25; p = 0.27).

Regarding cytokine quantification, the Δ-change observed in the probiotic group revealed an increase in IL-10 (+9.45; p = 0.12), IL-8 (+0.22; p = 0.35) and TNF-α (+0.77; p = 0.22). In addition, we observed a reduction in abdominal pain (p = 0.45), only suggested in this elaboration but more strongly supported by the subsequent data analysis.

No significant changes were observed in either symptoms or inflammation in the intra-group analyses.

Table 3 displays the results of analysis of data from the treatment and placebo groups over time which showed a statistically significant reduction in abdominal pain (−1.16; p < 0.05) and an increase in faecal IgA (+12.38; p < 0.05). These were the only two statistically significant results of the analyses.

**Table 3:** Difference between treatment and placebo.

Variable (unit)	Mean differences treatment A versus B	CI 95%	p value
Aerophagia	0.08	−0.92; 1.08	0.87
Diarrhea	0.17	−1.35; 1.39	0.98
Costipation	0.02	−1,35; 1,40	0.98
Abdominal pain	−1.16	−2.38; 0,04	**p**<**0.05**
Diarrhea-Constipation	−0.07	−1.12; 0.97	0.89
sIgA (μg/ml)	−1	−43.75; 41.76	0.96
sAA (ng/gr of feces)	7.75	−24.84; 40.33	0.62
CgA (pg/gr of feces)	130.30	−564.55; 825.13	0.71
Cortisol (ng/gr of feces)	0.41	−0.76; 1.58	0.48
IgA (μg/gr of feces)	12.38	−0.18; 1.35	**p**<**0.05**
IL8 (pg/gr of feces)	0.22	- 0.28; 0.73	0.38
IL10 (pg/gr of feces)	6.70	−30.63; 44.03	0.72
TNFa (pg/gr of feces)	0.21	−1.17; 1.58	0.77
NK 1:10 (%lysis)	−1.25	- 3.23; 0.73	0.21
NK 1:30 (%lysis)	−0.86	- 3.42; 1.70	0.51
NK 1:100 (%lysis)	−1.7	−5.20; 1.79	0.34

Bold characters highlight the statistically different values obtained from parameters investigation.

Table 4 shows the correlations between Δ-change (the difference between the beginning and end of the study) for each variable in the probiotic group. The correlations showed that reductions in aerophagia, abdominal pain and constipation were associated with an increase in IL-8 (p < 0.05). In addition, the increase in SAA appeared to be linked to an increase in IL-10 (r = 0.466; p < 0.05). A similar correlation was found between cortisol reduction and TNF increase (r = 0.311; p < 0.05).

**Table 4:** Pearson correlations between Δ-changes (t_1_-t_0_) of all markers following 45 days of treatment.

		Aerophagia	Diarrhea	Costipation	Abdominal pain	Diarrhea-Costipation	sigA	sAA	cgA	cortisol	IgA	IL8	IL10	TNFa	NK110	NK130	NK1100
Aerophagia	r.	1	−0,017	−0,257	0649	0,508	0084	0,392	−0,257	−0,206	0248	−0,515^∗^	0,206	0131	−0,339	−0,358	−0,236
Diarrhea	r.		1	−0,270	−0,118	−0,138	−0,237	−0,084	−0,169	0129	0,273	0195	−0,163	0113	0,071	−0,265	−0,197
Costipation	r.			1	**0,638**^∗^	**0,601**^∗^	0,127	−0,373	−0,335	−0,017	−0,293	**−0,619**^∗^	−0,021	−0,100	−0,101	−0,029	-,0018
Abdominal pain	r.				1	**,773**^∗∗^	,050	−0,141	−0,323	−0,264	0218	**−0,569**^∗∗^	0,133	0140	−0,066	−0,297	−0,192
Diarrhea-Costipation	r.					1	−0,118	0037	0,134	−0,225	0116	**−0,504**^∗^	-,012	0,052	0004	−0,078	−0,134
sIgA (μg/ml)	r.						1	−0,125	−0,031	0091	−0,012	−0,063	0148	−0,059	0025	0,206	0223
sAA (ng/gr of feces)	r.							1	0,148	−0,044	0192	0,059	**0,466**^∗∗^	−0,118	−0,244	−0,230	−0,301
CgA (pg/gr of feces)	r.								1	,183	0,034	0,563^∗∗^	0,057	**−0,513**^∗∗^	-,108	0,181	0143
Cortisol (ng/gr of feces)	r.									1	0,081	0090	0,246	**−0,311**^∗^	0,109	0203	0,181
IgA (μg/gr of feces)	r.										1	0,113	−0,190	-,058	−0,015	−0,203	−0,172
IL8 (pg/gr of feces)	r.											1	0,112	−0,164	0018	0,117	0173
IL10 (pg/gr of feces)	r.												1	-,040	0,053	0062	0,018
TNFa (pg/gr of feces)	r.													1	−0,049	−0,146	−0,147
NK 1:10 (%lysis)	r.														1	**0,439**^∗∗^	**0,392**^∗∗^
NK 1:30 (%lysis)	r.															1	**0,806**^∗∗^
NK 1:100 (%lysis)	r.																1

**Pearson's correlations are in the upper triangular matrix with P values (in **bold**: P < 0.05).

No statistically significant variations in microbiota composition following probiotic administration were detected.

Alpha diversity was calculated using the Chao I and Shannon indexes, but no significant difference was seen, indicating that intra-sample diversity was not particularly influenced by either the probiotic or the placebo.

When between-group and intra–group differences were examined, no significant changes in average bacterial group composition were seen following administration of the two products, even when the probiotic was compared with the placebo.

**Fig. 2:**
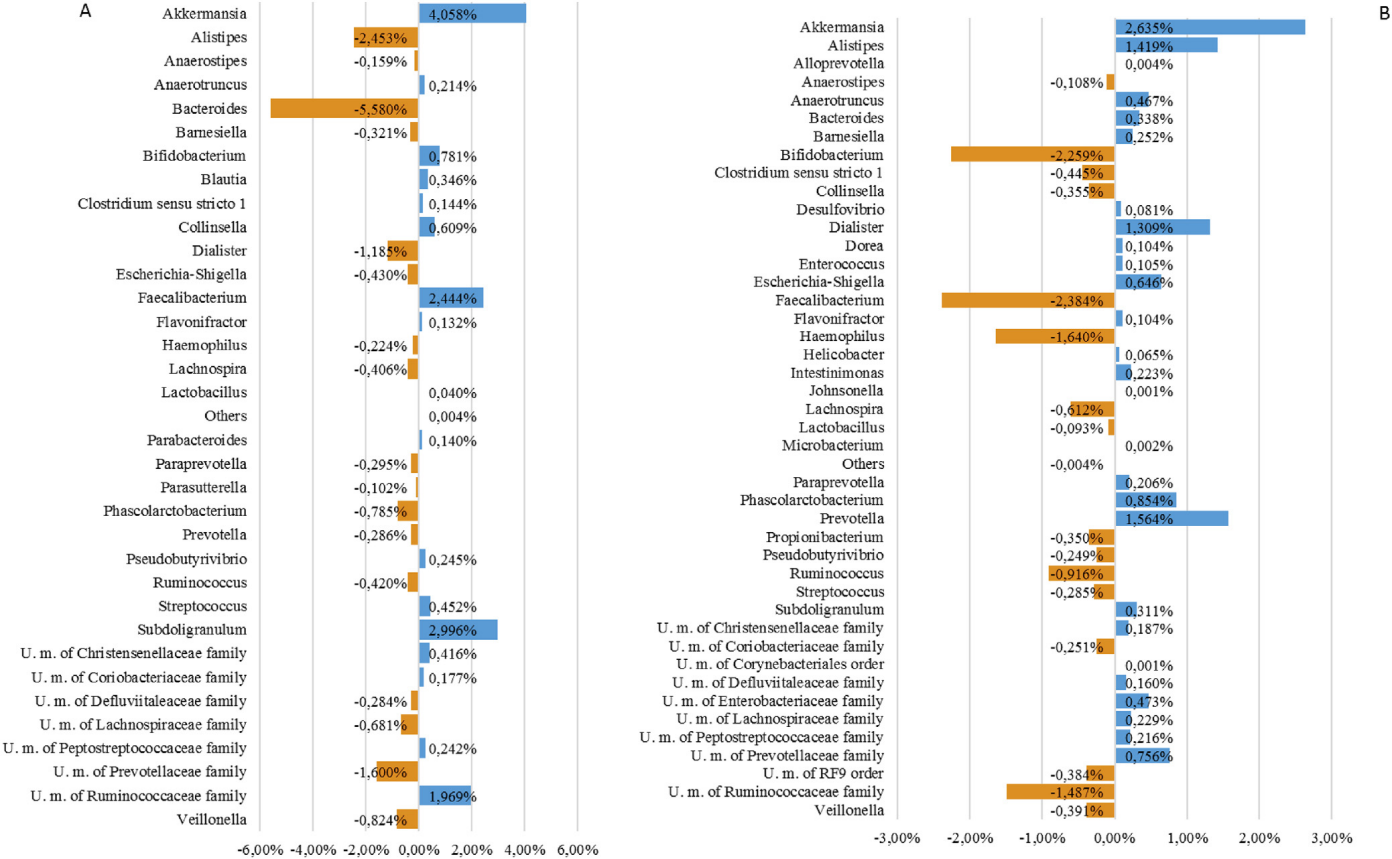
Graphical representation of changes that occurred during the study in the largest gut bacterial populations. (A) Average percentages of bacterial composition in the treatment group; (B) average percentages of bacterial composition in the placebo group.

Data obtained from the sequencing elaboration are displayed in Fig. 2, which shows the absolute percentage differences in the samples between T_0_ and T_45_. The bar chart summarises the increasing/decreasing taxa with a prevalence >80% (those taxa that have been identified in at least 80% of the samples considered in the comparison) and an absolute percentage difference >0.1%.

No statistically significant changes were seen between the beginning and the end of the study for either treatment, but some interesting trends were observed that suggest the probiotic mixture exerted an anti-inflammatory effect. When the probiotic is compared to placebo, Δ–change calculated for specific populations revealed the following: an increase of 3.04% and 0.13%, respectively, in *Bifidobacterium* and *Lactobacillus* spp., as expected; a decrease of 2.49% in *Dialister* spp., an increase of 4.83% in *Faecalibacterium* spp.; and a decrease of 1.08% in the *Escherichia* and *Shigella* group.

### 3.3 Adverse events

No serious adverse events (SAEs) were reported. However, seven subjects reported nine AEs, ranging from mild to moderate, none of which were judged to be related to the investigated product.

Eight subjects reported concurrent medication. Only one subject (code 2582) took a prohibited drug (Augmentin 1000 mg, twice daily) from 23 to 28 May 2106 during the wash-out period (from 17 May to 06 June 2016).

## 4 Discussion

The present study was designed to investigate whether a probiotic product administered to subjects experiencing stress-related symptoms could induce an improvement of the immunological markers associated to inflammation and negative somatic effects related to stress such as gut symptoms or possible microbiota imbalance. For a quantitative approach to the aim of the study we measured several immunological markers in blood, saliva and fecal water, being supported by previous works of many authors who have correlated these parameters to stress. On the other hand, possible effects exerted by the product on the fluctuations in gastrointestinal symptoms were investigated by the administration of a questionnaire.

As documented by many authors, an emerging application for probiotics is their use as psychobiotics to help treat mental disorders and stress/anxiety symptoms (Bambury et al., 2017; Foster et al., 2017; Misra and Mohanty, 2017).

One of the limitations of our study was the fact that at baseline subjects self-reported stress, which was not confirmed by serological parameters; however, the administration of questionnaires to identify eligible subjects also serves to screen volunteers in case of bias associated with self-assessed activity, as reported in a study conducted on stress symptoms by Fischer et al. (2016).

The lack of NK cell activity impairment meant a true stress condition could not be confirmed in subjects who did not show significant changes following product/placebo intake. This output was unexpected and three different problems could have influenced the result: the self – reported stressed condition of the subjects; patients’ individual characteristics, not directly linked to the study, but determined by intra and interindividual variability; the relatively small number of enrolled subjects.

Administration of the probiotic mixture to healthy volunteers with psychological stress did not directly affect stress salivary markers but did stimulate the immune system.

It was not possible to detect an overall effect on microbiota composition since microbial populations in healthy subjects are usually well balanced and, for this reason, intervention with probiotics is not expected to interfere dramatically in absence of specific or precise perturbations.

Fluctuations in different bacterial populations at the beginning and end of the study in both groups did not reach statistical significance but did suggest a trend, which was further supported by the immunological data.

The influence of the probiotic on *Bifidobacteria* and *Lactobacilli* populations was slight but, as explained above, probiotic administration will not always have clear effects in a stable environment.

The microbiological data suggest the probiotic supported anti-inflammatory bacteria which can protect the intestinal mucosa through SCFA production (*Faecalibacterium* spp.) and reduced pro–inflammatory populations, such as *Dialister* spp., which have been correlated with gut inflammation (Thorkildsen et al., 2013; Tito et al., 2017). This result was in agreement with the observational study of Jiang et al. (2015) in which patients with major depressive disorders showed reduced *Faecalibacterium* and increased *Enterobacteriaceae*, with a negative correlation between *Faecalibacterium* and the severity of depressive symptoms.

Comparison of subjects taking placebo with those taking the probiotic revealed that the placebo group showed an increase in potentially detrimental or pro-inflammatory bacteria, while subjects taking the probiotic did not.

Further supporting this effect, probiotic treatment resulted in a reduction in abdominal pain and an increase in IL-10 and faecal IgA levels. The faecal IgA increase implies the product supports mucosal immune efficiency. This result, consistent with microbiological observations, may reflect a stronger intestinal mucosa which is less prone to infections and colonisation by pathogenic bacteria, and agrees with the work of Bambling et al. (2017), Fukushima et al. (1998) and Nishida et al. (2017).

As demonstrated by Dong et al. (2012), *Bifidobacterium* strains are good inducers of IL-6, IL-10 and MCP-1. IL-10 is a regulatory cytokine which can inhibit the synthesis of other pro-inflammatory cytokines (IFN-γ, IL-2, IL-3, TNF-α and GM-CSF) produced by T helper lymphocytes (Th1). This cytokine down-regulates the inflammatory response and induces an antibody-mediated immune response. For these reasons, the recorded IL-10 increase is a positive result.

The probiotic strains tested, as demonstrated for *Lactobacillus* GG (Rautava et al., 2006) and *Saccharomyces boulardii* (Rodrigues et al., 2000), have been shown to enhance IgA production and secretion through changes in the cytokine milieu of the gut mucosa. Probiotic bacteria, in general, have been reported to induce epithelial cell expression of IL-10 and IL-6, supporting IgA production through B-cell maturation (He et al., 2007; Shang et al., 2008; Wells et al., 2017).

There are several examples in the literature of studies on the effects of probiotic modulation of the gut–brain axis. Results are often contradictory regarding the applicability of results obtained in animal models to humans: in a randomised, placebo-controlled study of healthy men and women, psychological stress and anxiety improved following intake of a *Lactobacillus*/*Bifidobacterium*-containing probiotic compared with taking a control product, but another study using a different *Lactobacillus* strain failed to confirm these findings (Benton et al., 2007; Kelly et al., 2017; Messaoudi et al., 2011).

Nevertheless, anti-inflammatory activity was consistently observed following the administration of probiotics in cases of mental disorder in animal models and in human trials (Bambling et al., 2017; Nishida et al., 2017; Vanhaecke et al., 2017; Vlainić et al., 2016). It has also been shown that treatment with the probiotic bacterium *Lactobacillus farciminis* attenuated the HPA stress response through prevention of intestinal barrier impairment (Ait-Belgnaoui et al., 2012; Bercik et al., 2010).

In our trial, the product's efficacy seemed to be more indirect, supporting and protecting the mucosal barrier against possible imbalance and lowering intestinal inflammation, as indicated by the symptom questionnaire which showed a decrease in abdominal discomfort.

Regarding the correlation between inflammation and mood/mental disorders, it has been established that pro-inflammatory cytokines induce neuropsychological symptoms in vulnerable individuals, suggesting that the brain–cytokine system is involved in depression (Dantzer et al., 2008; Vlainić et al., 2016).

The present work also showed increased faecal IgA immunoglobulins which play an important role in protecting mucosal surfaces against pathogens, fortifying the barrier for the compartmentalization of the bacteria in the lumen (Wells et al., 2017).

In light of the above, the anti-inflammatory trend and immunological stimulation displayed by the microbiota, and positively influenced by probiotic intake, could also be related to the decreased incidence in stressed subjects of common conditions typically related to stress, and their repercussions on physical health.

## Declaration of interest

1. Authors’ declaration of personal interests:

Ariella Annoni is an employee of Montefarmaco OTC. The other authors report no conflicts of interest.

## Funding

This research did not receive any specific grant from funding agencies in the public, commercial or not-for-profit sectors.

The probiotic strains *Lactobacillus acidophilus* LA-5^®^, *Bifidobacterium animalis* subsp. *lactis*, BB-12^®^ and *Lactobacillus paracasei* subsp. *paracasei*, L. CASEI 431^*®*^ are manufactured by Chr. Hansen A/S, Denmark. LA-5^®^, BB-12^®^ and L. CASEI 431^*®*^ are registered trademarks of Chr. Hansen A/S.

This work was supported by Montefarmaco OTC.

## Authorship

Guarantor of the article: Dr. Sara Soldi.

## CRediT authorship contribution statement

**Sara Soldi:** Investigation. **Sara Carlotta Tagliacarne:** Formal analysis. **Chiara Valsecchi:** Formal analysis. **Simone Perna:** Formal analysis. **Mariangela Rondanelli:** Formal analysis. **Luigi Ziviani:** Project administration, Writing - review & editing. **Stefano Milleri:** Project administration, Writing - review & editing. **Ariella Annoni:** Resources. **Annamaria Castellazzi:** Conceptualization.
