# Supplementary material for: Effect of a multistrain probiotic (Lactoflorene® Plus) on inflammatory parameters and microbiota composition in subjects with stress-related symptoms
Source: Neurobiol Stress. 2018 Nov 7;10:100138. doi: 10.1016/j.ynstr.2018.11.001 (PMC6430185; doi:10.1016/j.ynstr.2018.11.001)
Supplement: Multimedia component 1 [file mmc1.docx]

**SUPPORTING INFORMARTION**

Additional Supporting Information may be found online in the supporting information tab for this article.

**Complete list of inclusion/exclusion criteria:**

Inclusion criteria:

- Healthy adults (or with changes that the principal investigator or a physician deemed not to interfere with safety or study results), men and women aged between 20 and 35 years

- Men and women with a value of S.T.A.I. scale Y module (state anxiety) ≥ 35 and ≥40, respectively

- Last antibiotic treatment terminated at least 30 days before the start of the trial

- People able to properly carry out and complete the trial

- People who have read, understood and signed the informed consent form

Main Exclusion criteria:

- Intake of yogurt and products containing probiotics and prebiotics within 3 weeks before the start of the treatment

- Administration of antibiotics and / or laxatives in the last 30 days before the start of the study

- People with known chronic diseases gastrointestinal

- People who regularly take drugs for gastrointestinal diseases (eg laxatives, anti-diarrhea, digestive)

- People who regularly take medications for congenital gastrointestinal disease

- People with food allergies or intolerances

- People involved in any clinical study in the previous 6 months

**Administered symptoms’ questionnaire**

| Symptoms | Score |
| --- | --- |
| aerofagia | (score from -3 to 3) |
| diarrhea | (score from -3 to 3) |
| constipation | (score from -3 to 3) |
| abdominal pain | (score from -3 to 3) |
| diarrhea/constipation | (score from -3 to 3) |

-3 indicates an increase of the symptoms

0 indicates that no modification occurred

+3 indicates an amelioration of the symptoms
